# The Landscape of HNF1B Deficiency: A Syndrome Not Yet Fully Explored

**DOI:** 10.3390/cells12020307

**Published:** 2023-01-13

**Authors:** Alessandro Gambella, Silvia Kalantari, Massimiliano Cadamuro, Marco Quaglia, Maurizio Delvecchio, Luca Fabris, Michele Pinon

**Affiliations:** 1 Department of Medical Sciences, University of Turin, 10126 Turin, Italy; 2 Division of Liver and Transplant Pathology, University of Pittsburgh, Pittsburgh, PA 15232, USA; 3 Department of Molecular Medicine, University of Padova, 35121 Padua, Italy; 4 Department of Translational Medicine, University of Piemonte Orientale, 28100 Novara, Italy; 5 Metabolic Disease and Genetics Unit, Giovanni XXIII Children’s Hospital, AOU Policlinico di Bari, 70124 Bari, Italy; 6 Liver Center, Digestive Disease Section, Department of Internal Medicine, Yale University, New Haven, CT 06510, USA; 7 Pediatric Gastroenterology Unit, Regina Margherita Children’s Hospital, AOU Città della Salute e della Scienza di Torino, 10126 Turin, Italy

**Keywords:** HNF1B deficiency, non-neoplastic condition, tumor, MODY, kidney cyst, hepatopathy, cholestasis, cognitive impairment

## Abstract

The hepatocyte nuclear factor 1β (HNF1B) gene is involved in the development of specialized epithelia of several organs during the early and late phases of embryogenesis, performing its function mainly by regulating the cell cycle and apoptosis pathways. The first pathogenic variant of HNF1B (namely, R177X) was reported in 1997 and is associated with the maturity-onset diabetes of the young. Since then, more than 230 different HNF1B variants have been reported, revealing a multifaceted syndrome with complex and heterogenous genetic, pathologic, and clinical profiles, mainly affecting the pediatric population. The pancreas and kidneys are the most frequently affected organs, resulting in diabetes, renal cysts, and a decrease in renal function, leading, in 2001, to the definition of HNF1B deficiency syndrome, including renal cysts and diabetes. However, several other organs and systems have since emerged as being affected by HNF1B defect, while diabetes and renal cysts are not always present. Especially, liver involvement has generally been overlooked but recently emerged as particularly relevant (mostly showing chronically elevated liver enzymes) and with a putative relation with tumor development, thus requiring a more granular analysis. Nowadays, HNF1B-associated disease has been recognized as a clinical entity with a broader and more variable multisystem phenotype, but the reasons for the phenotypic heterogeneity are still poorly understood. In this review, we aimed to describe the multifaceted nature of HNF1B deficiency in the pediatric and adult populations: we analyzed the genetic, phenotypic, and clinical features of this complex and misdiagnosed syndrome, covering the most frequent, unusual, and recently identified traits.

## 1. HNF1B Deficiency: Genetics and Historical Background

Hepatocyte nuclear factors (HNFs) are transcriptional factors regulating tissue development and function of several organs. Overall, HNFs are classified into four groups based on their functional domains, namely HNF1, HNF3 (or FoxA), HNF4, and HNF6 [(or Onecut (OC)]. Notably, the HNF1 group, that includes *Hepatocyte nuclear factor 1α* (HNF1A) and *Hepatocyte nuclear factor 1β* [*HNF1B*, also known as *Transcription Factor 2 (TCF2)*], has been increasingly studied due to its association with disease [1]. In particular, heterozygous pathogenic variants in the *HNF1B* gene are the most commonly identified genetic cause of organ malformations in the pediatric populations, especially affecting kidney development. The gene is located on chromosome 17q12 and encodes hepatocyte nuclear factor 1β, a member of the homeodomain-containing family of transcription factors, which plays a key role in the morphogenesis of several organs, especially the kidney, pancreas, and liver [2].

The first *HNF1B* pathogenic variant (R177X) was described in a Japanese family with maturity-onset diabetes of the young (MODY), in 1997 [3]. MODY is a monogenic and autosomal dominant form of diabetes mellitus whose onset usually occurs before 25 years of age [4]. This disease is due to a dysfunction of pancreatic β cells characterized by non-ketotic diabetes and absence of pancreatic autoantibodies. Extrapancreatic manifestations are rarely found in the different subtypes of MODY and HNF1B-related disease is an exception in this regard [4]. The involvement of *HNF1B* pathogenic gene variants in renal disease was observed in pedigrees affected by MODY5: these patients often display renal cysts and renal function decline that preceded pancreatic dysfunction. The disease was then renamed renal cysts and diabetes syndrome in 2001 (RCAD, #137920) [5].

Nowadays, it is known that several other organs and systems can be affected by the perturbation of the *HNF1B* signaling, and that diabetes and renal cysts are not always present. Therefore, HNF1B-associated disease has been recognized as a clinical entity with a broader and more variable multi-system phenotype, which may be due to the functional promiscuity of the HNF1B transcription factor, as it regulates the development of the urogenital tract, brain, and parathyroid gland, in addition to the kidney, liver, and pancreas, by acting in concert with several other developmental genes [5].

HNF1B-associated disease expresses an autosomal dominant inheritance pattern; however, de novo pathogenic variants account for up to 50% of cases, and, therefore, a family history of the disease may be absent [6]. More than 230 different *HNF1B* allelic variants have been reported in association with disease, according to the Human Gene Mutation Database, including missense, nonsense, frameshift, and splicing mutations [7]. The most frequently identified variants are clustered in the first 4 exons of the gene, with exons 2 and 4, and the intron 2 splice site being hotspots [6]. There is no available evidence to suggest that patients with whole-gene deletions exhibit a different phenotype from those with pathogenic variants at the coding or splice site, suggesting that the dysfunctions are due to a gene dosage defect (i.e., haploinsufficiency) [8]. The most commonly identified genetic alteration (in approximately 50% of patients) is a complete gene deletion, in the context of 17q12 chromosomal microdeletion, which also includes 14 other genes [2]. Some disease-associated phenotypes, in particular the neurological ones, appear to occur only in the context of this microdeletion and, consequently, might not be directly linked to *HNF1B* itself [2].

To date, no clear correlation between the type or position of a pathogenic variant within *HNF1B* and the occurrence of particular clinical features has been clearly demonstrated [9]. The reasons for the phenotypic variation remain poorly understood and may reflect the functional effects of different gene anomalies, stochastic variation in temporal *HNF1B* gene expression during the earliest developmental stages, or additional genetic and/or environmental modifiers [3].

In particular, the extreme variability of *HNF1B* deficiency could be due to the deregulation or malfunction of proteins belonging to the signaling cascade of *HNF1B*. However, although several genes regulated by *HFN1B* have been identified using candidate gene approaches, e.g., cystic disease and cilia-related proteins, the full set of downstream genes that are responsible for the physiological and pathological functions of HFN1B remains elusive. In particular, the signaling cascade involved in the development of the liver phenotype is completely missing.

Additionally, pediatric and adult populations seem to be differently affected. Although the pediatric population has been more thoroughly studied, adult manifestations of HNF1B-deficiency start being more frequently observed, and kindle the need of a more granular analysis (Table 1).

In this Review, we performed an extensive analysis of the published available literature from 1997 (year of publication of the first published pathogenic variant of *HNF1B)* [3] to date (2022). We queried PubMed, Scopus, Embase, and Web of Science databases using the following keywords and MeSH (Medical Subject Headings) words: (“HNF1B” OR “HNF1β”) AND (“Hepatocyte nuclear factor”) AND (“HNF”) AND (“TCF2” OR “TCF-2”) AND (“Transcription Factor 2”). Pre-clinical (in vitro and in vivo animal models) and clinical studies were both considered, but non-English written papers were excluded. The title and abstract of the studies identified were then evaluated for appropriateness, and corresponding references were revised to grant literature research adequacy. From each study, details about study design and *HNF1B*-related data (with a particular focus on genetic, histopathologic, and clinical features) were critically analyzed and summarized.

## 2. Kidney Involvement in HNF1B Deficiency

### 2.1. Clinical Spectrum

Renal phenotype is predominantly characterized by a chronic tubulointerstitial pattern, with bland urinalysis in most patients, absence of hematuria, low-grade tubular proteinuria, and low prevalence of arterial hypertension. In this setting, a renal biopsy usually shows an interstitial fibrosis with enlarged glomeruli or oligomeganephronia and enlarged proximal and distal tubules [10,17]. These lesions are the consequence of impaired energetic homeostasis of the renal tubule due to *HNF1B* pathogenic variants [18]. Hypomagnesemia and hypokalemia are both frequent (62% and are 46%, respectively) and are often found even in advanced chronic kidney disease (CKD; stage III–V) [10]. The high prevalence of hypomagnesemia is confirmed also in other studies and is due to magnesium tubular wasting [12]. This defect is ascribed to the altered control of *FXYD2* gene expression by HNF1B [19], whereas potassium loss may be secondary to magnesium deficiency [20]. Of note, primary isolated hypomagnesemia can be the first clinical manifestation of HNF1B nephropathy [21] and genetic testing should be considered in this setting [22]. 

Some data are emerging on the mechanisms and progression trajectory of HNF1B nephropathy towards CKD and end-stage kidney disease (ESKD) [23]. Although the median decrease is reported to be around −2.45 mL/min/year, as expected in tubulointerstitial form, rapid unexplained worsening of renal function has been reported, and CKD is found in more than 90% of adult patients at diagnosis [10]. Dubois-Laforgue et al. reported a high prevalence of stages III and IV of CKD (44%) and even ESKD (21%) [12]. Interestingly, patients with 17q12 deletion appear to have CKD stage III–IV and ESKD less often at diagnosis and a significantly better renal function in the follow-up than patients with different pathogenic variants, suggesting a genotype/phenotype correlation [24]. Arterial hypertension, proteinuria, age at the time of diagnosis of diabetes, and the presence of microvascular complications (retinopathy and neuropathy) are also factors correlated with CKD progression. This group of risk factors may reflect the coexistence of diabetic nephropathy in a subset of patients, who are also at increased cardiovascular risk [12]. This “albuminuric pattern” (as opposed to the more common one with low-grade tubular proteinuria) seems to be characterized by a more rapid functional deterioration in the long-term [25]. 

Renal structural abnormalities (RSA) are another key feature of HNF1B nephropathy and display significant heterogeneity. *HNF1B* pathogenic variants putatively account for ∼10% of congenital abnormalities in patients with kidney and urinary tract, both among children and adults [13]. A cystic phenotype is frequently observed, with most adult patients harboring less than 5 cortical cysts. These usually spare the kidney outline, do not determine an increase in kidney size, and do not increase in number over time. No correlation has been found between cyst development and renal function. While this course differentiates HNF1B nephropathy from Autosomal Dominant Polycystic Kidney Disease (ADPKD), a few cases of massive cysts mimicking ADPKD have also been reported [10]. Around 20% of patients showed single-kidney disease that can represent the evolution of a multicystic and dysplastic contralateral kidney. A wide range of other RSAs and renal manifestations have been reported, including hydronephrosis or hydroureter, vesical ureteral reflux, kidney stones, and nephrocalcinosis [10].

The clinical characteristics of HNF1B nephropathy in adults have been investigated mainly in 2 multicentric retrospective studies. In the study by Faguer et al., data of 27 adult patients (mean age: 35 years) were collected and analyzed [10]. Although 4/27 patients were symptom-free (14.8%), in the remaining 23 patients, the most common first manifestation was related to kidney involvement (61%), followed by diabetes (9%), genital tract malformations (18%), and liver test abnormalities (12%) [10]. The more recent and consistent study by Dubois-Laforgue et al. included 201 patients aged 18 or older with a high prevalence of diabetes (82%) and genital tract abnormalities (50% in female and 80% in male) [12]. HNF1B nephropathy is probably underdiagnosed among adult patients with CKD of an unknown cause, even in presence of compatible RSA. The sequencing of *HNF1B* gene in a cohort of unrelated adult patients with unknown etiology and RSA allowed the identification of *HNF1B* pathogenic variants in a subset of 9% of patients [14].

### 2.2. Histopathology of Non-Neoplastic Conditions

The kidney is by far one of the most commonly affected organs in HNF1B syndrome. Although diabetes (in the form of MODY-5) is also frequently observed in HNF1B-deficient patients, the kidney involvement is not related to diabetic dysfunction (i.e., diabetic nephropathy), but rather to an abnormal embryonic development [26]. 

In line with the key role played by HNF1B in kidney development, the most common structural alteration causing renal dysfunction is related to nephron dysgenesis [26,27,28]. In a rodent model of kidney embryonal development, the HNF1B transcript increased, starting from the early stages of polarized epithelium differentiation, and was maintained until complete maturation of the nephron [28]. Similar findings were also observed in a mouse model, further confirming the involvement of HNF1B in early nephrogenesis [29]. Furthermore, its expression was mostly restricted to the tubular compartment (proximal and distal tubules, and collecting ducts), while the glomeruli and the urothelial system appeared only partially affected [27,28,30,31,32]. This broad involvement in nephron development being specifically expressed by the tubular compartment, explains cystic disease as the main histopathological findings associated to HNF1B syndrome [26]. 

Additional functions of HNF1B in kidney physiology (such as tubular transport and organ metabolism) can justify the entire spectrum of renal pathological conditions observed. In particular, patients with HNF1B deficiency typically presented renal hypoplasia and agenesia, familial juvenile hyperuricemic nephropathy, glomerulocystic kidney disease, and renal interstitial fibrosis, eventually leading to chronic kidney disease [23,31,33,34,35,36,37,38,39].

### 2.3. Histopathology of Neoplastic Conditions

In addition to the regulation of organ development during embryogenesis, HNF1B also controls the expression of genes involved in cell cycle regulation and apoptosis, thereby making its assessment also relevant for neoplastic conditions [40,41,42]. Notably, HNF1B seems to be more related to the development of tumors with “clear cell” features, but its specific pathogenetic mechanism remains largely unexplored and thus, ambiguous [43,44,45,46,47]. 

Focusing on kidney neoplastic disease, the development of kidney tumors in the context of HNF1B deficiency syndrome (with or without underlying kidney disease) was rarely observed and reported [48,49,50]. In contrast, the expression of HNF1B *per se* in kidney tumors has been widely explored and its immunohistochemical evaluation was proposed to differentiate renal oncocytoma (diffusely and strongly positive, nuclear stain) from chromophobe renal cell carcinoma (negative) [51,52,53,54,55]. In clear cell renal cell carcinoma, down-regulation of HNF1B was mainly observed, and its loss was associated with high-grade tumor and metastatic disease, suggesting both tumor-suppressive functions and prognostic potential [43,49], as also shown in Wilms tumor [56,57]. Conversely, papillary renal cell carcinoma, mucinous tubular spindle cell carcinoma, and metanephric adenoma were characterized by HNF1B overexpression, supporting its role as a proto-oncogene [58,59]. Overall, these apparently contradictory findings confirm the multifaceted role of HNF1B in kidney tumor development and support the need for broader analysis and validation studies.

**Figure 1:**
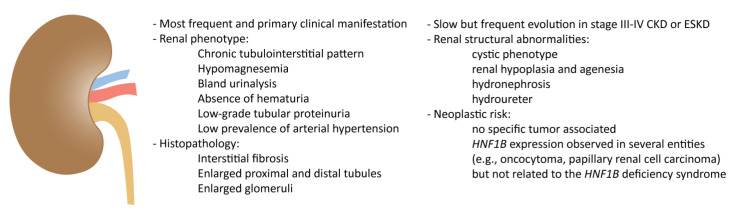
The *HNF1B* deficiency and the kidney.

The main features of the kidney involvement in HNF1B deficiency are summarized in Figure 1.

## 3. Liver Involvement in HNF1B Deficiency

### 3.1. Clinical Spectrum 

Although *HNF1B* defect is probably one of the most frequently recognized monogenic causes of developmental renal disease, abnormalities in liver function tests are frequently reported (around 40% in adults), especially in association with MODY5, where they reach 85% of cases [34]. Nevertheless, due to the initial description of HNF1B-deficiency as a syndrome characterized by kidney disease and diabetes, the liver involvement in *HNF1B* deficiency has been frequently overlooked, and only a few studies reported a comprehensive evaluation of the hepatic disease phenotypes [60]. Therefore, liver involvement has been poorly investigated, and reported so far as asymptomatic increase in transaminase levels or, less frequently, as a cholestatic liver disease.

A cholestatic condition featuring high gamma-glutamyl transferase (GGT) levels was reported in 5 newborns, who were all small for their gestational age (SGA), with a history of intrauterine growth restriction (IUGR), and affected by multiple bilateral renal cysts and moderate renal function impairment. Diabetes requiring insulin therapy occurred at an average age of 10 years in 3/5 cases, while 2/5 showed pancreatic hypoplasia with impaired pancreatic exocrine function [60,61,62,63,64]. Moreover, our group has recently described an additional case of a child with high GGT-cholestasis due to a de novo missense pathogenic variant of *HNF1B*, thus implicating rare mutations in *HNF1B* in the pathogenetic role in cholestatic liver diseases with increased GGT (Table 2) [65,66]. 

Genes causing low or high GGT cholestatic liver disease have either hepatocyte expression or cholangiocyte expression, respectively. Based on the current evidence, HNF1B has been included among the genetic causes of high GGT cholestasis derived from a cholangiocyte dysfunction, in both children and adults [63]. 

Interestingly, a similar clinical/histological liver phenotype was observed in 13 children from 9 unrelated consanguineous families with high GGT cholestatic liver disease, all presenting homozygous damaging variants in kinesin family member 12 (KIF12; Table 3) [64,65], a target gene known to be regulated by the HNF1B transcription factor [62].

KIF12 belongs to the large kinesin superfamily of microtubule-associated molecular motors, which are crucial in the intracellular transport of organelles, microtubule cytoskeleton organization, and mitotic spindle function for cell division.

Since the knowledge of genetic cholestasis is continuously evolving, several transporters, cell–cell junctional proteins, adaptive processes, and intracellular signaling pathways involved in normal hepatobiliary homeostasis and bile acid metabolism may be regarded as new potential candidate genes for cholestasis.

Among them, within the new classification of progressive familial intrahepatic cholestasis (PFIC) [68], *KIF12* pathogenic variants have been included as driving mutations responsible for PFIC-8. PFIC-8 pediatric patients are characterized by increased serum levels of GGT and transaminases, hyperbilirubinemia, hypercholesterolemia, pruritus, and by a rapid development of bridging fibrosis associated with ductopenia. Of note, recent data demonstrated that KIF12 is under the control and downstream effector of HNF1B. This transcription factor regulates KIF12 expression in both cultured cells, and knockout mice, by altering co-factor recruitment and histone modification, which supports a potential role of KIF12 in the function of cholangiocytes [70,71]. It is currently unclear which are the mechanisms linking these two types of chronic cholestasis and whether they can depend on common molecular bases. Considering the mutual interactions between KIF12 and HNF1B and the overlapping clinical profile, we speculate that *HNF1B* pathogenic variants should also be considered as part of the PFIC spectrum. A recent work by Stalke et al. has shown that, in 6 pediatric patients harboring KIF12 variants, cholestasis may be due to a perturbed polarization of hepatocytes, which leads to an incorrect positioning of the hepatocanalicular membrane of channel proteins, such as ATP binding cassette subfamily B member (ABCB) 4 and ABCB11, regulating the transport and extrusion of bile salts into the bile canaliculus [69]. Similarly, patients with *HNF1B* pathogenic variants, whose malfunction can negatively regulate KIF12 expression, may present a similar derangement of the correct exposure of canalicular transporters. Future research directions will help understanding the pathogenesis of PFIC-8 and its relationship with HNF1β-related cholestasis, hopefully unveiling novel targets for therapeutic intervention. In keeping with this observation, ileal bile acid transporter (IBAT) inhibitors, have been proposed for the treatment of patients with PFIC and Alagille syndrome, and approved in mid-2021 to treat intractable pruritus related to cholestasis [72,73,74]. Moreover, these new drugs already applied to other types of PFIC and cholestatic liver disease [69], could be considered in children and adults with KIF12 and HNF1B defective function. Indeed, although genetic cholestatic liver diseases are more frequently observed in children, novel genetic alterations are continuously identified thanks to high-throughput genetic techniques, thereby providing candidate gene variants that may apply to some causes of unknown cholestasis in adults. In addition, further research may provide new insights on the complex mechanisms that combine cholestasis, hepatic repair/regenerative response, and biliary fibrosis, which could also be relevant for acquired cholestasis, according to the hypothesis that genetic diseases can serve as a ‘pathophysiological roadmap’ to improve the understanding of other pathogenic factors [75].

### 3.2. Histopathology of Non-Neoplastic Conditions

As HNF1B is involved in the development of specialized epithelia, its dysfunction in the liver is primarily related to the bile duct system. In fact, mouse models harboring liver-specific HNF1B inactivation are characterized by abnormal development of gallbladder and intrahepatic bile ducts, coupled with their respective interlobular arteries, clinically resulting in jaundice and failure to thrive [76,77,78]. The liver of these mice showed histopathological alterations (i.e., senescence alterations and epithelial metaplasia) in the gallbladder and intrahepatic bile ducts. Additionally, hepatic ductal plate remnants were still observed in adult mice [76,77,78]. This evidence is particularly relevant, as it supports the notion that HNF1B is key in the development of the bile duct epithelia, in both extra- and intrahepatic systems. Furthermore, ciliogenesis and cell polarization were found to be primarily and severely impaired [79,80,81], defining the HNF1B-related dysmorphogenic biliary phenotype. These defects may have an underlying developmental origin coming from anomalies in ductal plate remodeling, resulting in ductal plate malformations (DPMs), with the persistence of post-natal embryonic biliary structures, biliary cell clusters or duct-like structures [82]. According to this hypothesis, *HNF1B* may behave as a pivotal regulator of primitive ductal structures (PDS). Indeed, in a new pathogenic classification, DPMs are not the result of a lack of PDS remodeling, but rather the common endpoint of different defects of differentiation, maturation, expansion, polarity and/or ciliogenesis of PDS, affecting distinct stages of bile duct morphogenesis. For example, mice with *HNF1B* deficiency showed a normal hepatic differentiation, but an abnormal PDS maturation (73). The cilium hosts many proteins that can sense physical and chemical properties of the biliary milieu, and its dysfunction is associated with increased cholangiocyte proliferation and profound changes in cholangiocyte intracellular signaling. The role of HNF1B in ciliogenesis emerged with ultrastructural analysis of 3 adults with late-onset cholestasis, demonstrating a significant loss of primary cilia in cholangiocytes, but no structural intra- or extrahepatic bile duct defects [15]. Based on this evidence, HNF1B deficiency is included in the wide spectrum of syndromic ciliopathies with liver involvement [83]. 

The involvement of the extrahepatic bile ducts secondary to HNF1B deficiency has been rarely documented, and mainly reported as the complete absence [60] or (choledochal) cystic degeneration, with atypical morphology on imaging studies [84]. Kettunen et al. described biliary abnormalities, identified by magnetic resonance (MR) cholangiography, in 6 patients with *HNF1B* mutations. Most of them had varying types of bile duct cysts (BDCs) in the extrahepatic bile ducts, with an atypical morphology for any given Todani classification.

On this basis, it is not surprising that the liver phenotype of patients with HNF1B deficiency shows a cholestatic profile [15,60,61,62,63,85,86], especially in the neonatal age. From a histopathological perspective, all the cholestatic patients reported above [60,61,62,63] had comparable histological features, showing a pattern of paucity of interlobular bile ducts (PILBD), associated with marked cholestasis and variable degrees of periportal fibrosis. Clinical PILBD is not such a rare finding at histology, especially in infants with cholestasis and accounts for the 11% of pediatric liver biopsies [87]. PILBD has been categorized as syndromic (S-PILBD) and non-syndromic (NS-PILBD) entities. S-PILBD is associated with Alagille Syndrome (AGS), whereas a source of diagnostic dilemma to clinicians is the presence of ductopenia in patients who otherwise do not fit the clinical AGS description or are non-syndromic. The paucity of bile ducts is in fact not pathognomonic for AGS and can be found in several disorders of different etiologies, as shown in Table 4. 

When these additional clinical traits are absent, PILBDs require a broad diagnostic work-up, needing supplementary testing and characterization. Therefore, the identification of HNF1B deficiency as a possible cause of PILBD partially addresses the question regarding bile duct paucity in patients who do not meet the criteria for AGS, the most frequent cause of PILBD. In accordance with these findings, it has recently been stated that a ciliopathy like HNF1B deficiency must be regarded as a new condition to be included in the diagnostic work-up of neonatal/infantile cholestasis, when PILBD is observed at liver biopsy and other features of AGS are absent [66].

A defective HNF1B function may also play a role in the pathogenesis of neonatal sclerosing cholangitis (NSC) similarly to patients with *DCDC2* (doublecortin domain containing protein 2) pathogenic variants. To date, 13 patients with NSC have been reported [89,90,91]. They show high GGT cholestasis, acholic stool, and progression to portal hypertension with radiological findings revealing typical structuring with dilatations in the intrahepatic and/or extrahepatic biliary tree. Renal and neurological abnormalities were also frequently present. Liver histology showed peripheral ductopenia, DPM, fibrosis, and eventually, cirrhosis. Interestingly, *DCDC2* has been shown to be closely related to the ciliary kinesin-2 subunit *KIF3a*. However, cases of HNF1B deficiency with a less prominent involvement of the bile duct system were also reported, showing no significant characteristics or steatotic/steatohepatitic changes, mainly in adolescent/adult patients [15,16,34,61,85]. 

Altogether, these observations pinpoint HNF1B-deficiency as a significant cause of liver involvement, especially in the neonatal/pediatric settings, ultimately suggesting HNF1B to be included in the diagnostic algorithm of patients presenting with a cholestatic profile [66]. 

### 3.3. Histopathology of Neoplastic Conditions

The association of HNF1B deficiency with the development of primary liver cancer is largely unexplored. Single nucleotide polymorphisms (SNPs) of *HNF1B* have been identified and associated through genotyping arrays with cancer development. *HNF1B* somatic mutations were observed in several human cancers, among which hepatocellular carcinoma (HCC), confirming further, as previously discussed, its role as an oncogene/tumor suppressor gene [50]. Nevertheless, the association of HCC with germline *HNF1B* deficiency is largely uncharted [6,65]. However, although at a very low risk, *HNF1B* deficiency may be associated with HCC, as in some PFIC disorders. Interesting enough, although *HNF1B* deficiency mainly affects the biliary epithelium, only HCC but not cholangiocarcinoma, has been observed so far. HCC is the second most common malignant liver tumor in children after hepatoblastoma. Both hepatoblastoma and HCC account together for the 0.5–1.5% of all childhood malignancies and for the 4% of all pediatric liver transplantations [92]. It differs from adult HCC with respect to the etiological predisposition, biological behavior, and lower frequency of cirrhosis. *HNF1A* mutations occur in 1–2% of HCC.

HCC was firstly reported in HNF1B deficiency in a 16-month-old newborn with a germline pathogenic variant in *HNF1B*, presenting renal hyperechogenicity, transient renal neonatal failure, and progressive neonatal cholestasis, ultimately evolving to micronodular cirrhosis and HCC. According to this report, the little patient underwent liver transplantation with no relapse (1 year of follow-up) [64]. Additionally, a report described a 9-month-old girl with ductopenic cholestasis, hyperparathyroidism, growth retardation, and delayed motor development who developed HCC featuring a syncytial giant cell subtype [93]. Notably, the perilesional liver showed injured bile ducts or complete bile duct loss, similar to AGS. Although HNF1B deficiency was not considered in the differential diagnosis, we hint at the possibility that this HCC could be a misdiagnosed case of HNF1B deficiency. Similarly, we described a case of HNF1B deficiency affected by chronic renal disease due to multicystic kidney involvement, bilateral cryptorchidism, and autism spectrum disorder [94]. Noteworthy, our patient also developed a syncytial giant cell subtype of HCC, which led us to hypothesize that the syncytial giant cell variant of pediatric HCC could be strictly related to the HNF1B faulty-driven oncogenetic mechanism, as it has never been reported elsewhere [95,96,97,98]. 

Although not yet unraveled, HNF1B deficiency-dependent mechanisms promoting HCC could be secondary to its repressive effect on *HNF1A* transcriptional activity, since HNF1B forms a heterodimer with HNF1A and binds to the same target elements as investigated by Kitanaka et al. [63]. We can therefore speculate that the phenotypic variability of patients with *HNF1B* pathogenic variants might be caused by different HNF1B activity in conjunction with repression of HNF1A activity, in selected promoters and tissues. Further functional studies on these effects are sorely needed to clarify this issue. From this perspective, we are generating both an animal model of HNF1B-KO mouse and mouse organoids to understand the pathophysiology of liver disease associated with perturbations in the HNF1B signaling pathway (EASL Daniel Alagille Award 2021).

**Figure 2:**
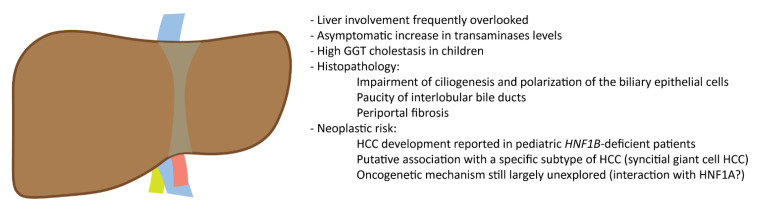
The *HNF1B* deficiency and the liver.

The main features of the liver involvement in HNF1B deficiency are summarized in Figure 2.

## 4. Pancreas Involvement in HNF1B Deficiency

### 4.1. Clinical Spectrum

Pancreatic atrophy is reported in approximately 30% of patients and diabetes mellitus in about 50%. Diabetes mellitus is the most common extrarenal feature identified in patients with HNF1B-associated disease, mostly developing as a typical MODY, which is a form of diabetes mellitus secondary to pancreatic beta cell dysfunction, with a typical onset before 25 years of age, the absence of specific anti-beta cell antibodies, and autosomal dominant inheritance. This subtype of MODY is usually known as MODY5 or HNF1B/MODY, according to the most recent nomenclature.

HNF1B/MODY represents up to 6% of all MODY cases and is one of the most frequent causes of syndromic diabetes [99,100], showing a decrease in insulin secretion with progressive worsening of blood glucose control. The severity and course of diabetes are variable and glucose homeostasis ranges from normoglycemia to insulin-treated diabetes with ketoacidosis at onset [101]. Hyperglycemia usually occurs during adolescence or early adulthood [6], when there is a physiological increase in insulin resistance. Hyperglycemia can rarely occur early during the neonatal age [35,102]. The presence of decreased insulin secretion and increased insulin resistance lead to irreversible chronic hyperglycemia, which appears similar to other forms of HNFs/MODY. In the study of Faguer et al., almost 70% of patients required insulin in the follow-up, but no case of retinopathy and neuropathy was reported [34], whereas in the experience of Dubois-Laforgue et al., microvascular complications were detected in 40% of patients after a 15 year-observation period [12]. 

Extrapancreatic features are evident before the onset of diabetes, and the presence of CKD at the onset of diabetes is a typical feature [101]. Indeed, patients with HNF1B-deficient syndrome rarely show only diabetes and this diagnosis should be suspected at diabetes onset in all the patients with renal cysts and/or other suggestive extra-pancreatic features, even when a family history is absent.

Of note, patients with HNF1B nephropathy who undergo renal transplant are at increased risk of developing post-transplant diabetes mellitus (PTDM) [10]. Therefore, HNF1B defects should be considered when PTDM develops after kidney transplantation [103,104], and should also be ruled out also in the absence of any apparent risk factor, particularly in a young kidney transplant recipient with compatible features and an unknown causal nephropathy [105]. Calcineurin inhibitor treatment after transplant seems to reduce the expression of the wild-type allele of *HNF1B* gene, leading to insufficient transcriptional activity. The evidence that carriers of the HNF1B deficiency are at a greater risk for diabetes mellitus raised the question of whether transplant management must be modified to avoid drugs that can cause hyperglycemia. In this light, immunosuppressive agents other than corticosteroids should be preferred. Therefore, a tailored immunosuppressive regimen could be considered in HNF1B patients [106]. Finally, diabetic patients with HNF1B nephropathy reaching end-stage renal disease (ESRD) are a potential candidate for simultaneous pancreatic and kidney transplantation.

Regarding the treatment of HNF1B/MODY, patients present hepatic insulin resistance to some extent [107], and thus the treatment with oral hypoglycemic agents such as sulfonylureas may not be satisfactory, and early insulin therapy may be requested [108]. In the large study cohort described by Dubois-Laforgue et al. [12], 49% of the patients were treated with insulin since diabetes onset, but the rate increased to 79% during the follow-up because of the worsening glucose control. In a small subgroup of patients, the treatment with oral hypoglycemic agents (sulphonylurea or meglitinide repaglinide) was started after a mean time of 8 months from diagnosis and it was successful in 57% of patients. In these patients, HbA1c dropped from 7.1% to 6.1% and the beta-cell function result improved after 5 years of treatments. A case report described the switch from insulin to glimepiride after reaching excellent metabolic control. Sitagliptin was added during follow-up, allowing excellent glucose control for 6 years. After this period, the blood glucose control worsened and insulin treatment was necessary [109]. 

Chronic complications are poorly described in the literature, even if it is likely that they may occur as frequently as in other types of diabetes and are related to glycemic control [12]. However, long-term data are missing. Similarly, data on care during pregnancy are not available in the literature, but insulin treatment is usually necessary to keep blood glucose within normal limits. Birth weight is normal, as long as maternal hyperglycemia is properly treated, but if the fetus carriers HNF1B-deficiency, the birth weight is typically low [35].

### 4.2. Histopathology of Non-Neoplastic Condition

HNF1B plays a key role in the early development and differentiation of the pancreas by regulating the expression of proteins related to the organ embryogenesis, such as HNF4A and SLC2A2, and variations in *HNF1B* gene activity can impact both the exocrine and endocrine compartments. Indeed, beta-cells are another cell type where HNF1B drives embryological development and functionality, as demonstrated by *Hnf1B*-knock out mice models showing hypoplastic and atrophic pancreas, as well as beta-cell impaired terminal differentiation [110,111]. Similar findings were also observed in HNF1B-deficient fetuses and patients [112,113], leading to the well-described MODY-5 clinical phenotype [3,34,112,113,114]. However, most significant changes in pancreas morphology described so far are based on imaging studies [CT/MRI of the pancreas and magnetic resonance cholangiopancreatography (MRCP)], while only a few studies report histopathological changes [34,113,115,116]. Based on imaging description, the pancreas showed no pancreatic body and tail, and the accessory and dorsal ducts were lacking as well, whereas the beta-cell impairment was mainly based on clinical, functional, and serological evaluations. In contrast to the extrahepatic bile duct involvement reported above, no cystic dilatations were reported. Overall, these findings suggested a predominant involvement/agenesis of the dorsal pancreatic component, but the ventral bud (and the head of the pancreas), although mostly spared, also appeared to be partially involved [3,34,113,115,116]. It is noteworthy that parenchymal calcifications were also reported, but not constantly and without a correlation with patient condition or pancreatic morphology, although an association with late-phase dysfunction following diabetes development has been suggested [113]. 

Histopathological analysis of *HNF1B*-mutated fetal pancreatic tissue confirmed imaging studies, showing a severe hypoplasia of the pancreatic body and tail, further suggesting its cause as severe acinar component underdevelopment. Indeed, most patients with HNF1B-related pancreatic hypoplasia show a subclinical pancreatic exocrine dysfunction, assessed through fecal elastase deficiency [6]. Furthermore, Langerhans islets were morphologically disorganized and beta cells slightly reduced [112]. 

### 4.3. Histopathology of Neoplastic Condition

The role of HNF1B in pancreatic cancer development was mainly related to tumor suppressor functions [50,117]. Indeed, several studies performed in different models of pancreatic ductal adenocarcinoma (including organoids, cell cultures, mice models, and human tissue samples) demonstrated an overall suppressed expression of HNF1B, as genomic transcript or immunohistochemical nuclear stain, compared to the pancreatic acinar parenchyma and ducts [117,118,119,120]. Similarly, down-regulation of HNF1B was observed in mouse models and organoids of intraductal papillary mucinous neoplasms [120,121].

**Figure 3:**
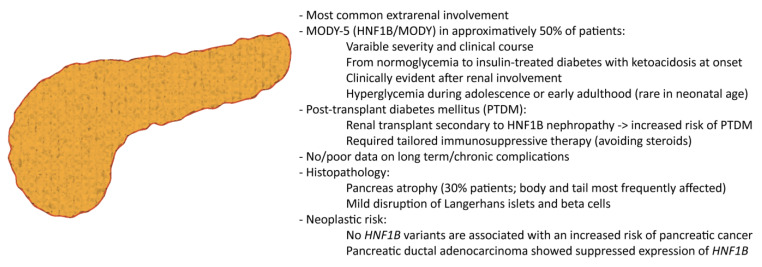
The *HNF1B* deficiency and the pancreas.

The mechanism of tumor development related to reduced HNF1B levels appeared to be related to a hypermethylation of the promoter region, eventually leading to: (i) loss of the adhesion molecule E-cadherin in neoplastic cells, (ii) expanded epithelial-to-mesenchymal transition, and (iii) increased tumor cell migration [50,117]. However, no variants of *HNF1B* have been hitherto associated with an increased risk of pancreatic cancer development [122]. The main features of pancreatic involvement in HNF1B deficiency are summarized in Figure 3.

## 5. Additional Extrarenal Involvement

Genital tract abnormalities are found in around 40% of female patients, usually presenting major and severe malformations (e.g., bicornuate or rudimentary uterus, vaginal aplasia (Müllerian aplasia)) that were also observed in the context of the Mayer-Rokitansky-Küster-Hauser syndrome [10,123,124]. However, the exact prevalence is most likely largely underestimated due to the presence of minor abnormalities with no/minor clinical significance that could potentially be overlooked [10,123,124]. Notably, *HNF1B* pathogenic variants were found in 18% of patients with structural alterations in both the kidney and the uterus, but were not found in isolated uterine malformations [125]. Male genital defects, such as cryptorchidism, abnormal descent of testes, hypospadias, and prostatic hypoplasia, were also reported [126]. 

Mild cognitive impairment, seizures, autism, schizophrenia, and structural alterations of the brain have only been reported in patients with deletions of chromosome 17q12 [127,128,129,130]. However, mild neurological involvement is frequently observed and deserves a more thorough analysis, especially in the adult population [10]. 

Finally, anecdotal abnormalities and malformations of other districts were also reported, including facial dysmorphic features [131], duodenal atresia [131], prune belly syndrome (also known as Eagle-Barrett syndrome) [126,132], congenital diaphragmatic hernia [133], and congenital joint laxity [134].

Considering this complex landscape, two types of scores have been proposed to select patients for genetic screening based on clinical criteria [13,135]. In the latter, the coexistence of cysts of unknown origin and hypomagnesemia determines a high screening priority, as it is associated with a 6-fold increased probability of finding HNF1B-related pathogenic variants.

## 6. Conclusions

HNF1B-associated disease has been recognized as a clinical entity with a broader and more variable multisystem phenotype than previously reported. Despite the frequent and peculiar involvement of the kidneys and pancreas, HNF1B deficiency has increasingly emerged as a multifaceted syndromic ciliopathy affecting several organs, among which the liver seems to be quite relevant. The consequent complex and heterogeneous clinical phenotype, most likely related to the functional interaction of *HNF1B* with other developmental genes, could impinge on patient management. We believe that this comprehensive review, the first providing a broad overview of the genetic, histopathologic, and clinical phenotypes of HNF1B-deficiency syndrome, will kindle a more detailed analysis of the effect of *HNF1B* pathogenic variants and the related clinical landscape. Joint efforts from multidisciplinary research groups are then required to foster the understanding of the pathophysiology and molecular mechanisms of this highly complex syndrome, with the aim to identify clinically relevant implications, that may translate the evolving pediatric knowledge to adult clinicians.

## Figures and Tables

**Table 1:** List of the main studies evaluating the clinical manifestations of HNF1B mutations in the adult population.

Patients with *HNF1B* Pathogenic Variants	Mean Age	Main Findings	Reference
27	35	- Most frequent renal phenotype: chronic tubulointerstitial nephritis, cystic phenotype, and chronic renal failure; - Hypomagnesemia and hypokalemia (despite chronic kidney disease);	[10]
28	24	- Point mutations and large genomic rearrangements presented similar clinical phenotype;	[11]
201	>18	- High prevalence of diabetes (82%) and genital tract abnormalities (50% in female and 80% in male);	[12]
8	34.8	- Cysts unknown origin and hypo/dysplasia as major criteria of congenital anomalies of kidneys and urinary tract; - HNF1B genetic analysis to patients having bilateral major renal anomalies regardless the age at presentation;	[13]
6	23	- Relatively high prevalence of HNF1B mutations in adult patients with chronic kidney disease of unknown etiology; - Typical but subtle signs of renal involvement present for many years before diagnosis;	[14]
3	38.7	- First evidence of absence or paucity of primary cilia on bile duct epithelial cells as the cause of significant cholestasis in adult patients with HNF1B-deficiency;	[15]
4	43.7	- Chronic increase of liver enzymes (biliary phenotype) in a family with HNF1B mutation; - First report of adult patient with HNF1B mutation without diabetes;	[16]

**Table 2:** Clinical and histopathological features of patients’ with HNF1B variants presenting with neonatal cholestasis.

Gender/Origin GW/BW g (DS)	Liver Involvement	Liver Histology	Renal Function and Ultrasound Findings	Pancreatic Involvement	Growth	Urogenital Malformations/ Cognitive Impairment	HNF1B Variant	Reference
♂/Japan 39/2390 (−2.26) <1 month	- High GGT neonatal cholestasis, acholic stools - No abnormality of extrahepatic bile ducts at explorative surgery - Cholestasis resolution at 9-month follow-up with persistent mild transaminases alteration - Transient hypercholesterolemia	PILBD, marked cholestasis	- Multiple bilateral cysts (right, four 1–2 cm diameter cysts, left, one 1 cm diameter cyst) - Mild chronic renal insufficiency	Diabetes requiring insulin therapy at 13 years of age (polyuria and polydipsia, mild metabolic acidosis)	NA	Absent/mild	c.457C>A, p.H135N (missense mutation in exon 2, de novo or paternal: history of liver dysfunction and renal insufficiency in his paternal family)	[63]
♂/Belgium (Sardinian origin) 37/1520 (−3.46) <1 month	- High GGT neonatal cholestasis, slightly enlarged liver - Cholestasis resolution at 1-year follow-up with persistent mild transaminases alteration - 3 episodes of cholangitis - Hypertriglyceridemia (300 mg/dL)	PILBD, severe biliary stasis, slight periportal fibrosis	- Left kidney agenesis, enlarged and hyperechogenic right kidney, multiple cortical cysts - Progressive chronic renal failure from 19 months	- Diabetes requiring insulin therapy at 5 years of age without ketoacidosis - Pancreatic atrophy with progressive exocrine pancreatic deficiency requiring enzyme substitution from the age of 16 years	Final height of 162.1 cm (−1.86 SD), BMI 19.0 kg/m^2^ (−0.62 SD)	Absent/NA	499–504 delGCTCTG insCCCCT, A167FS (combination of a deletion and insertion in exon 2, de novo)	[62]
♂/Germany 35/1780 (−1.69) <1 month	- High GGT neonatal cholestasis, acholic stools - Cholestasis resolution at 1 year follow-up with persistent mild transaminases alteration - Hypercholesterolemia (292 mg/dL) and hypertriglyceridemia (307 mg/dL)	PILBD	- Severe bilateral kidney malformations (cystic kidney dysplasia and hydronephrosis due to urethral stenosis) - Chronic renal insufficiency	- Diabetes requiring insulin therapy at 13 years of age - Pancreatic hypoplasia with progressive exocrine pancreatic deficiency	Final height of 133.9 cm (−6.7 SD), BMI 17.3 kg/m^2^ (−2.1 SD)	Inguinal hernia, abdominal testis/delayed psychomotor development	*HNF1B* deletion exons 1–9, de novo	[61]
♀/Czech Republic 38/2360 (−1.60) <1 month	- High GGT neonatal cholestasis, acholic stools - Kasai portoenterostomy at 32 days of age based on lack of visualization of extrahepatic bile ducts at explorative surgery - Progressive increase in liver function tests, mainly cholestatic - Multiple cysts in the left hepatic lobe (diameter from 2 to 7 mm)	PILBD, cholestasis without signs of bile duct proliferation	- Multiple bilateral cortical cysts (maximal diameter 5 mm), prenatally hyperechogenic kidneys - Normal renal function by 2-year follow-up - Mild hypomagnesemia	- Pancreatic hypoplasia (absent body and tail) without exocrine pancreatic deficiency - Normoglycemia by 2-year follow-up	Growth along the 3rd centile	Absent/absent	1698 kb deletion including *HNF1B*, de novo	[60]
♂/France 35/NA <1 month	- High GGT neonatal cholestasis without acholic stools - Hepatocellular carcinoma with elevated alpha-fetoprotein levels at 16 months of age requiring liver transplant - No relapse at 1-year follow-up	- Multinodular hepatic tumor and micronodular cirrhosis at the explant - No information available on PILBD	- Renal hyperechogenicity - Transient renal failure	NA	NA	NA/NA	1.5 Mb deletion including *HNF1B*	[64]
♂/Italy 38/2600 (−1.27) <1 month	- High GGT neonatal cholestasis, hypocholic stools - Persistent cholestasis and pruritus at 40-month follow-up - Hypercholesterolemia (256 mg/dL) and hypertriglyceridemia (120 mg/dL)	PILBD, biliary stasis	- Hyperechogenic kidneys, with multiple bilateral cortical cysts (maximum size 2 mm) - Chronic renal insufficiency	Initial pancreatic exocrine dysfunction without pancreatic hypoplasia at US	Growth along the 10th centile	Absent/absent	c.827G>A, p.R276Q (missense mutation in exon 4, de novo)	[65]

NA: information not available, GW: gestation weeks, BW: birth weight, PILBD: paucity of intralobular bile ducts, BMI: body mass index, US: ultrasound.

**Table 3:** Clinical and histopathological features of patients with KIF12 variants presenting with neonatal cholestasis.

Gender/Origin GW/BW g (DS) Age of Presentation	Liver Involvement	Liver Histology	Renal Function and Ultrasound Findings	Pancreatic Involvement	Growth	Urogenital Malformations/ Cognitive Impairment	*KIF12* Homozygous Variant (NM_138424.1)	Reference
(A) ♀/Syrian (consanguineous) full term 1 month	- High GGT neonatal cholestasis - Hypercholesterolemia - Persistent cholestasis at 22-month follow-up	PILBD, bridging fibrosis with early nodule formation, mixed portal inflammatory infiltrate, multinucleated giant hepatocytes, extensive hepatocanalicular cholestasis, ductular reaction	Left hydronephrosis and mild increase in left renal pelvic anterior–posterior diameter to 6 mm	NA	NA	NA	c.655C>T: p. (Arg219 *)	[67]
(B) ♂/Turkish (consanguineous) full term 2 months	- High GGT cholestasis at 2 months - Hypercholesterolemia - MRI: no biliary dilatation, strictures, or other liver lesions - Persistent cholestasis at 21-month follow-up	PILBD, biliary pattern of cirrhosis with nodule formation, mixed portal inflammatory infiltrate, mild ductular reaction, pseudo-acini formation, and nodule formation	Right renal pelvic anterior–posterior diameter	NA	NA	NA	c.610G>A: p. (Val204Met)	[67]
(C) ♂/Turkish (consanguineous) 9 years; ibling of patient B	- High GGT prolonged jaundice - MRI: no biliary dilatation, strictures, or other liver lesions - Not available follow-up	Not performed	Caliectasis of the upper pole of the left kidney	NA	NA	NA	c.610G>A: p. (Val204Met)	[67]
(D) ♂/4 months	- High GGT cholestasis - Persistent cholestasis at 5-year follow-up	NA	Normal	Absent	Normal	Absent	c.463C>T: p. (Arg155*)	[68]
(E) ♂/Unknown (consanguineous)/5 years	- High GGT cholestasis - LTx at 6 years	NA	Normal	Absent	Normal	Absent	c.656G>A: p. (Arg219Gln)	[68]
(F) ♂/Unknown (consanguineous)/14 months	- High GGT cholestasis sclerosing cholangitis - Persistent cholestasis at 11-year follow-up	Suggestive of biliary cirrhosis	Normal	Absent	Normal	Absent	c.610G>A: p. (Val204Met)	[68]
(G) ♂/Unknown (consanguineous)/6 months	- High GGT cholestasis - LTx at 10 months	Suggestive of biliary atresia	Normal	Absent	Normal	Absent	c.610G>A: p. (Val204Met)	[68]
(E) ♀/Kurdish (consanguineous)/ 13 years	- High GGT cholestasis - Liver cirrhosis - LTx at the age of 12y	Extensive liver fibrosis, only minimal inflammation and proliferated bile ducts	Normal	Absent	Normal	Absent	c.655C>T: p. (Arg219 *)	[69]
(F) ♂/Kurdish (consanguineous)/ 13 years	- High GGT cholestasis	Advanced fibrosis and bile duct proliferation	Normal	Pancreatic lipomatosis	Normal	Absent	c.655C>T: p. (Arg219 *)	[69]
(G) ♀/Iraqi (consanguineous)/ 7 years	- Neonatal cholestasis	NA	Normal	Absent	Normal	Absent	c.655C>T: p. (Arg219 *)	[69]
(H) ♀/Iraqi (consanguineous)/ 5 years	- Neonatal cholestasis - Progressive liver cirrhosis	Canalicular cholestasis, moderate fibrosis, mild inflammation without steatosis	Normal	Absent	Failure to thrive	Absent	c.655C>T: p. (Arg219 *)	[69]
(I) ♂/Syrian (consanguineous)/12 years	- High GGT cholestasis	Cirrhosis, septal hepatitis and ductular proliferation	Normal	Absent	Normal	Absent	c.655C>T: p. (Arg219 *)	[69]
(J) ♀/Afghan (consanguineous)/10 years	- Neonatal cholestasis - LTx at 4 years	NA	Normal	Absent	Normal	Absent	c.482-4_500del p. ?	[69]

NA: information not available, GW: gestation weeks, BW: birth weight, PILBD: paucity of intralobular bile ducts, BMI: body mass index, US: ultrasound. *: stop codon

**Table 4:** Paucity of bile ducts and associated disorders.

Genetic Disorders		Congenital Infections	Immune Disorders	Drug Related
AGS (OMIM # 118450, OMIM # 610205)	HNF1B deficiency syndrome (OMIM # 137920)	Cytomegalovirus	Sclerosing cholangitis	Vanishing bile duct syndrome
Cystic fibrosis (OMIM # 219700)	*KIF12*-associated cholestasis (OMIM # 619662)	Rubella	Hemophagocytic lymphohistiocytosis	
α1-antitrypsin deficiency (OMIM # 613490)	*ABBC12*-associated cholestasis [88]	Syphilis		
Niemann Pick type C (OMIM # 257220)				
Williams-Beuren syndrome (OMIM # 194050)				
Trisomy 21 (OMIM # 190685)				

## Data Availability

Not applicable.

## Abbreviations

AGS	Alagille syndrome
BDCs	bile duct cysts
BMI	body mass index
BW	birth weight
CKD	chronic kidney disease
DCDC2	doublecortin domain containing protein 2
DPMs	ductal plate malformations
EASL	European Association for the Study of the Liver
ESKD	end-stage kidney disease
HCC	hepatocellular carcinoma
HNF	hepatocyte nuclear factor
HNF1B	hepatocyte nuclear factor 1β
IBATis	ileal bile acid transporter inhibitors
IUGR	intrauterine growth restriction
KIF12	kinesin family member 12
MeSH	Medical Subject Headings
NA	information not available
NSC	neonatal sclerosing cholangitis
PDS	primitive ductal structures
PFIC	progressive familial intrahepatic cholestasis
PILBD	paucity of intralobular bile ducts
RSA	renal structural abnormalities
SGA	small for gestational age
SNPs	single nucleotide polymorphisms
TCF2	transcription factor 2
US	ultrasound

## References

[1] Lau H.H., Ng N.H.J., Loo L.S.W., Jasmen J.B., Teo A.K.K. (2018). The molecular functions of hepatocyte nuclear factors—In and beyond the liver. J. Hepatol..

[2] Bockenhauer D., Jaureguiberry G. (2016). HNF1B-associated clinical phenotypes: The kidney and beyond. Pediatr. Nephrol..

[3] El-Khairi R., Vallier L. (2016). The role of hepatocyte nuclear factor 1beta in disease and development. Diabetes Obes. Metab..

[4] Nyunt O., Wu J.Y., McGown I.N., Harris M., Huynh T., Leong G.M., Cowley D.M., Cotterill A.M. (2009). Investigating maturity onset diabetes of the young. Clin. Biochem. Rev..

[5] Verhave J.C., Bech A.P., Wetzels J.F., Nijenhuis T. (2016). Hepatocyte Nuclear Factor 1beta-Associated Kidney Disease: More than Renal Cysts and Diabetes. J. Am. Soc. Nephrol..

[6] Clissold R.L., Hamilton A.J., Hattersley A.T., Ellard S., Bingham C. (2015). HNF1B-associated renal and extra-renal disease-an expanding clinical spectrum. Nat. Rev. Nephrol..

[7] Stenson P.D., Mort M., Ball E.V., Chapman M., Evans K., Azevedo L., Hayden M., Heywood S., Millar D.S., Phillips A.D. (2020). The Human Gene Mutation Database (HGMD((R))): Optimizing its use in a clinical diagnostic or research setting. Hum. Genet..

[8] Pal A., Reidy K.J. (2017). Genetic Syndromes Affecting Kidney Development. Results Probl. Cell Differ..

[9] Mitchel M.W., Moreno-De-Luca D., Myers S.M., Levy R.V., Turner S., Ledbetter D.H., Martin C.L. 17q12 Recurrent Deletion Syndrome. https://www.ncbi.nlm.nih.gov/books/NBK401562/2020.

[10] Faguer S., Decramer S., Chassaing N., Bellanné-Chantelot C., Calvas P., Beaufils S., Bessenay L., Lengelé J.P., Dahan K., Ronco P. (2011). Diagnosis, management, and prognosis of HNF1B nephropathy in adulthood. Kidney Int..

[11] Bellanné-Chantelot C., Clauin S., Chauveau D., Collin P., Daumont M., Douillard C., Dubois-Laforgue D., Dusselier L., Gautier J.F., Jadoul M. (2005). Large genomic rearrangements in the hepatocyte nuclear factor-1beta (TCF2) gene are the most frequent cause of maturity-onset diabetes of the young type 5. Diabetes.

[12] Dubois-Laforgue D., Cornu E., Saint-Martin C., Coste J., Bellanné-Chantelot C., Timsit J., Monogenic Diabetes Study Group of the Société Francophone du Diabète (2017). Diabetes, Associated Clinical Spectrum, Long-term Prognosis, and Genotype/Phenotype Correlations in 201 Adult Patients with Hepatocyte Nuclear Factor 1B (HNF1B) Molecular Defects. Diabetes Care.

[13] Raaijmakers A., Corveleyn A., Devriendt K., van Tienoven T.P., Allegaert K., Van Dyck M., van den Heuvel L., Kuypers D., Claes K., Mekahli D. (2015). Criteria for HNF1B analysis in patients with congenital abnormalities of kidney and urinary tract. Nephrol. Dial. Transplant..

[14] Musetti C., Quaglia M., Mellone S., Pagani A., Fusco I., Monzani A., Giordano M., Stratta P. (2014). Chronic renal failure of unknown origin is caused by HNF1B mutations in 9% of adult patients: A single centre cohort analysis. Nephrology.

[15] Roelandt P., Antoniou A., Libbrecht L., Van Steenbergen W., Laleman W., Verslype C., Van der Merwe S., Nevens F., De Vos R., Fischer E. (2012). HNF1B deficiency causes ciliary defects in human cholangiocytes. Hepatology.

[16] Montoli A., Colussi G., Massa O., Caccia R., Rizzoni G., Civati G., Barbetti F. (2002). Renal cysts and diabetes syndrome linked to mutations of the hepatocyte nuclear factor-1 beta gene: Description of a new family with associated liver involvement. Am. J. Kidney Dis..

[17] Sagen J.V., Bostad L., Njolstad P.R., Sovik O. (2003). Enlarged nephrons and severe nondiabetic nephropathy in hepatocyte nuclear factor-1beta (HNF-1beta) mutation carriers. Kidney Int..

[18] Piedrafita A., Balayssac S., Casemayou A., Saulnier-Blache J.S., Lucas A., Iacovoni J.S., Breuil B., Chauveau D., Decramer S., Malet-Martino M. (2021). Hepatocyte nuclear factor-1beta shapes the energetic homeostasis of kidney tubule cells. FASEB J..

[19] Adalat S., Woolf A.S., Johnstone K.A., Wirsing A., Harries L.W., Long D.A., Hennekam R.C., Ledermann S.E., Rees L., Van′t Hoff W. (2009). HNF1B mutations associate with hypomagnesemia and renal magnesium wasting. J. Am. Soc. Nephrol..

[20] Yang L., Frindt G., Palmer L.G. (2010). Magnesium modulates ROMK channel-mediated potassium secretion. J. Am. Soc. Nephrol..

[21] Van Der Made C.I., Hoorn E.J., De La Faille R., Karaaslan H., Knoers N.V., Hoenderop J.G., Poussou R.V., De Baaij J.H. (2015). Hypomagnesemia as First Clinical Manifestation of ADTKD-HNF1B: A Case Series and Literature Review. Am. J. Nephrol..

[22] Musetti C., Quaglia M., Stratta P., Giordano M. (2015). Hypomagnesemia and progressive chronic kidney disease: Thinking of HNF1B and other genetic nephropathies. Kidney Int..

[23] Chan S.C., Zhang Y., Shao A., Avdulov S., Herrera J., Aboudehen K., Pontoglio M., Igarashi P. (2018). Mechanism of Fibrosis in HNF1B-Related Autosomal Dominant Tubulointerstitial Kidney Disease. J. Am. Soc. Nephrol..

[24] Ge S., Yang M., Cui Y., Wu J., Xu L., Dong J., Liao L. (2022). The Clinical Characteristics and Gene Mutations of Maturity-Onset Diabetes of the Young Type 5 in Sixty-One Patients. Front. Endocrinol..

[25] Hua Tan C.S., Ang S.F., Yeoh E., Goh B.X., Loh W.J., Shum C.F., May Ping Eng M., Yan Lun Liu A., Wan Ting Chan L., Goh L.X. (2022). MODY5 Hepatocyte Nuclear Factor 1ss (HNF1ss)-Associated Nephropathy: Experience from a regional monogenic diabetes referral centre in Singapore. J. Investig. Med. High Impact Case Rep..

[26] Bingham C., Ellard S., Allen L., Bulman M., Shepherd M., Frayling T., Berry P.J., Clark P.M., Lindner T., Bell G.I. (2000). Abnormal nephron development associated with a frameshift mutation in the transcription factor hepatocyte nuclear factor-1 beta. Kidney Int..

[27] Horster M.F., Braun G.S., Huber S.M. (1999). Embryonic renal epithelia: Induction, nephrogenesis, and cell differentiation. Physiol. Rev..

[28] Lazzaro D., De Simone V., De Magistris L., Lehtonen E., Cortese R. (1992). LFB1 and LFB3 homeoproteins are sequentially expressed during kidney development. Development.

[29] Lokmane L., Heliot C., Garcia-Villalba P., Fabre M., Cereghini S. (2010). vHNF1 functions in distinct regulatory circuits to control ureteric bud branching and early nephrogenesis. Development.

[30] Lindström N.O., McMahon J.A., Guo J., Tran T., Guo Q., Rutledge E., Parvez R.K., Saribekyan G., Schuler R.E., Liao C. (2018). Conserved and Divergent Features of Human and Mouse Kidney Organogenesis. J. Am. Soc. Nephrol..

[31] Ferre S., Igarashi P. (2019). New insights into the role of HNF-1beta in kidney (patho)physiology. Pediatr. Nephrol..

[32] Naylor R.W., Davidson A.J. (2014). Hnf1beta and nephron segmentation. Pediatr. Nephrol..

[33] Heidet L., Decramer S., Pawtowski A., Morinière V., Bandin F., Knebelmann B., Lebre A.S., Faguer S., Guigonis V., Antignac C. (2010). Spectrum of HNF1B mutations in a large cohort of patients who harbor renal diseases. Clin. J. Am. Soc. Nephrol..

[34] Bellanné-Chantelot C., Chauveau D., Gautier J.F., Dubois-Laforgue D., Clauin S., Beaufils S., Wilhelm J.M., Boitard C., Noël L.H., Velho G. (2004). Clinical spectrum associated with hepatocyte nuclear factor-1beta mutations. Ann. Intern. Med..

[35] Edghill E.L., Bingham C., Ellard S., Hattersley A.T. (2006). Mutations in hepatocyte nuclear factor-1beta and their related phenotypes. J. Med. Genet..

[36] Bingham C., Bulman M.P., Ellard S., Allen L.I., Lipkin G.W., van′t Hoff W.G., Woolf A.S., Rizzoni G., Novelli G., Nicholls A.J. (2001). Mutations in the hepatocyte nuclear factor-1beta gene are associated with familial hypoplastic glomerulocystic kidney disease. Am. J. Hum. Genet..

[37] Rizzoni G., Loirat C., Levy M., Milanesi C., Zachello G., Mathieu H. (1982). Familial hypoplastic glomerulocystic kidney. A new entity?. Clin. Nephrol..

[38] Kaplan B.S., Gordon I., Pincott J., Barratt T.M. (1989). Familial hypoplastic glomerulocystic kidney disease: A definite entity with dominant inheritance. Am. J. Med. Genet..

[39] Mache C.J., Preisegger K.H., Kopp S., Ratschek M., Ring E. (2002). De novo HNF-1 beta gene mutation in familial hypoplastic glomerulocystic kidney disease. Pediatr. Nephrol..

[40] Hojny J., Michalkova R., Krkavcova E., Bui Q.H., Bartu M., Nemejcova K., Kalousova M., Kleiblova P., Dundr P., Struzinska I. (2022). Comprehensive quantitative analysis of alternative splicing variants reveals the HNF1B mRNA splicing pattern in various tumour and non-tumour tissues. Sci. Rep..

[41] Suzuki E., Kajita S., Takahashi H., Matsumoto T., Tsuruta T., Saegusa M. (2015). Transcriptional upregulation of HNF-1beta by NF-kappaB in ovarian clear cell carcinoma modulates susceptibility to apoptosis through alteration in bcl-2 expression. Lab. Invest..

[42] Tsuchiya A., Sakamoto M., Yasuda J., Chuma M., Ohta T., Ohki M., Yasugi T., Taketani Y., Hirohashi S. (2003). Expression profiling in ovarian clear cell carcinoma: Identification of hepatocyte nuclear factor-1 beta as a molecular marker and a possible molecular target for therapy of ovarian clear cell carcinoma. Am. J. Pathol..

[43] Buchner A., Castro M., Hennig A., Popp T., Assmann G., Stief C.G., Zimmermann W. (2010). Downregulation of HNF-1B in renal cell carcinoma is associated with tumor progression and poor prognosis. Urology.

[44] Köbel M., Kalloger S.E., Carrick J., Huntsman D., Asad H., Oliva E., Ewanowich C.A., Soslow R.A., Gilks C.B. (2009). A limited panel of immunomarkers can reliably distinguish between clear cell and high-grade serous carcinoma of the ovary. Am. J. Surg. Pathol..

[45] Amano Y., Mandai M., Yamaguchi K., Matsumura N., Kharma B., Baba T., Abiko K., Hamanishi J., Yoshioka Y., Konishi I. (2015). Metabolic alterations caused by HNF1beta expression in ovarian clear cell carcinoma contribute to cell survival. Oncotarget.

[46] Cuff J., Salari K., Clarke N., Esheba G.E., Forster A.D., Huang S., West R.B., Higgins J.P., Longacre T.A., Pollack J.R. (2013). Integrative bioinformatics links HNF1B with clear cell carcinoma and tumor-associated thrombosis. PLoS ONE.

[47] Němejcová K., Tichá I., Kleiblová P., Bártů M., Cibula D., Jirsová K., Dundr P. (2016). Expression, Epigenetic and Genetic Changes of HNF1B in Endometrial Lesions. Pathol. Oncol. Res..

[48] Lebrun G., Vasiliu V., Bellanné-Chantelot C., Bensman A., Ulinski T., Chrétien Y., Grünfeld J.P. (2005). Cystic kidney disease, chromophobe renal cell carcinoma and TCF2 (HNF1 beta) mutations. Nat. Clin. Pract. Nephrol..

[49] Bártů M., Hojný J., Hájková N., Michálková R., Krkavcová E., Hadravský L., Kleissnerová L., Bui Q.H., Stružinská I., Němejcová K. (2020). Analysis of expression, epigenetic, and genetic changes of HNF1B in 130 kidney tumours. Sci. Rep..

[50] Chandra S., Srinivasan S., Batra J. (2021). Hepatocyte nuclear factor 1 beta: A perspective in cancer. Cancer Med..

[51] An J., Park C.K., Kim M., Joo J.W., Cho N.H. (2021). HNF-1beta as an immunohistochemical marker for distinguishing chromophobe renal cell carcinoma and hybrid oncocytic tumors from renal oncocytoma. Virchows Arch..

[52] Kato N., Motoyama T. (2009). Hepatocyte nuclear factor-1beta(HNF-1beta) in human urogenital organs: Its expression and role in embryogenesis and tumorigenesis. Histol. Histopathol..

[53] Wang C.C., Mao T.L., Yang W.C., Jeng Y.M. (2013). Underexpression of hepatocyte nuclear factor-1beta in chromophobe renal cell carcinoma. Histopathology.

[54] Conner J.R., Hirsch M.S., Jo V.Y. (2015). HNF1beta and S100A1 are useful biomarkers for distinguishing renal oncocytoma and chromophobe renal cell carcinoma in FNA and core needle biopsies. Cancer Cytopathol..

[55] Sun M., Tong P., Kong W., Dong B., Huang Y., Park I.Y., Zhou L., Liu X.D., Ding Z., Zhang X. (2017). HNF1B Loss Exacerbates the Development of Chromophobe Renal Cell Carcinomas. Cancer Res..

[56] Liu Y., Kanyomse Q., Xie Y. (2019). Tumor-suppressive activity of Hnf1beta in Wilms’ tumor. Biosci. Biotechnol. Biochem..

[57] Bártů M., Dundr P., Němejcová K., Tichá I., Hojný H., Hájková N. (2018). The Role of HNF1B in Tumorigenesis of Solid Tumours: A Review of Current Knowledge. Folia Biol..

[58] Szponar A., Yusenko M.V., Kuiper R., van Kessel A.G., Kovacs G. (2011). Genomic profiling of papillary renal cell tumours identifies small regions of DNA alterations: A possible role of HNF1B in tumour development. Histopathology.

[59] Banyai D., Sarlos D.P., Nagy A., Kovacs G. (2018). Recalling Cohnheim’s Theory: Papillary Renal Cell Tumor as a Model of Tumorigenesis from Impaired Embryonal Differentiation to Malignant Tumors in Adults. Int. J. Biol. Sci..

[60] Kotalova R., Dusatkova P., Cinek O., Dusatkova L., Dedic T., Seeman T., Lebl J., Pruhova S. (2015). Hepatic phenotypes of HNF1B gene mutations: A case of neonatal cholestasis requiring portoenterostomy and literature review. World J. Gastroenterol..

[61] Raile K., Klopocki E., Holder M., Wessel T., Galler A., Deiss D., Muller D., Riebel T., Horn D., Maringa M. (2009). Expanded clinical spectrum in hepatocyte nuclear factor 1b-maturity-onset diabetes of the young. J. Clin. Endocrinol. Metab..

[62] Beckers D., Bellanne-Chantelot C., Maes M. (2007). Neonatal cholestatic jaundice as the first symptom of a mutation in the hepatocyte nuclear factor-1beta gene (HNF-1beta). J. Pediatr..

[63] Kitanaka S., Miki Y., Hayashi Y., Igarashi T. (2004). Promoter-specific repression of hepatocyte nuclear factor (HNF)-1 beta and HNF-1 alpha transcriptional activity by an HNF-1 beta missense mutant associated with Type 5 maturity-onset diabetes of the young with hepatic and biliary manifestations. J. Clin. Endocrinol. Metab..

[64] de Leusse C., De Paula A.M., Aschero A., Parache C., Hery G., Cailliez M., Missirian C., Fabre A. (2019). Hepatocarcinoma and Cholestasis Associated to Germline Hemizygous Deletion of Gene HNF1B. J. Pediatr. Gastroenterol. Nutr..

[65] Pinon M., Carboni M., Colavito D., Cisarò F., Peruzzi L., Pizzol A., Calosso G., David E., Calvo P.L. (2019). Not only Alagille syndrome. Syndromic paucity of interlobular bile ducts secondary to HNF1beta deficiency: A case report and literature review. Ital. J. Pediatr..

[66] Mandato C., Zollo G., Vajro P. (2019). Cholestatic jaundice in infancy: Struggling with many old and new phenotypes. Ital. J. Pediatr..

[67] Ünlüsoy Aksu A., Das S.K., Nelson-Williams C., Jain D., Özbay Hoşnut F., Evirgen Şahin G., Lifton R.P., Vilarinho S. (2019). Recessive Mutations in KIF12 Cause High Gamma-Glutamyltransferase Cholestasis. Hepatol. Commun..

[68] Maddirevula S., Alhebbi H., Alqahtani A., Algoufi T., Alsaif H.S., Ibrahim N., Abdulwahab F., Barr M., Alzaidan H., Almehaideb A. (2019). Identification of novel loci for pediatric cholestatic liver disease defined by KIF12, PPM1F, USP53, LSR, and WDR83OS pathogenic variants. Genet. Med..

[69] Stalke A., Sgodda M., Cantz T., Skawran B., Lainka E., Hartleben B., Baumann U., Pfister E.D. (2022). KIF12 Variants and Disturbed Hepatocyte Polarity in Children with a Phenotypic Spectrum of Cholestatic Liver Disease. J. Pediatr..

[70] Gong Y., Ma Z., Patel V., Fischer E., Hiesberger T., Pontoglio M., Igarashi P. (2009). HNF-1beta regulates transcription of the PKD modifier gene Kif12. J. Am. Soc. Nephrol..

[71] Mrug M., Li R., Cui X., Schoeb T.R., Churchill G.A., Guay-Woodford L.M. (2005). Kinesin family member 12 is a candidate polycystic kidney disease modifier in the cpk mouse. J. Am. Soc. Nephrol..

[72] Kamath B.M., Stein P., Houwen R.H.J., Verkade H.J. (2020). Potential of ileal bile acid transporter inhibition as a therapeutic target in Alagille syndrome and progressive familial intrahepatic cholestasis. Liver Int..

[73] Karpen S.J., Kelly D., Mack C., Stein P. (2020). Ileal bile acid transporter inhibition as an anticholestatic therapeutic target in biliary atresia and other cholestatic disorders. Hepatol. Int..

[74] Gonzales E., Hardikar W., Stormon M., Baker A., Hierro L., Gliwicz D., Lacaille F., Lachaux A., Sturm E., Setchell K.D. (2021). Efficacy and safety of maralixibat treatment in patients with Alagille syndrome and cholestatic pruritus (ICONIC): A randomised phase 2 study. Lancet.

[75] Fabris L., Fiorotto R., Spirli C., Cadamuro M., Mariotti V., Perugorria M.J., Banales J.M., Strazzabosco M. (2019). Pathobiology of inherited biliary diseases: A roadmap to understand acquired liver diseases. Nat. Rev. Gastroenterol. Hepatol..

[76] Coffinier C., Gresh L., Fiette L., Tronche F., Schutz G., Babinet C., Pontoglio M., Yaniv M., Barra J. (2002). Bile system morphogenesis defects and liver dysfunction upon targeted deletion of HNF1beta. Development.

[77] Yamasaki H., Sada A., Iwata T., Niwa T., Tomizawa M., Xanthopoulos K.G., Koike T., Shiojiri N. (2006). Suppression of C/EBPalpha expression in periportal hepatoblasts may stimulate biliary cell differentiation through increased Hnf6 and Hnf1b expression. Development.

[78] Clotman F., Libbrecht L., Gresh L., Yaniv M., Roskams T., Rousseau G.G., Lemaigre F.P. (2003). Hepatic artery malformations associated with a primary defect in intrahepatic bile duct development. J. Hepatol..

[79] Raynaud P., Tate J., Callens C., Cordi S., Vandersmissen P., Carpentier R., Sempoux C., Devuyst O., Pierreux C.E., Courtoy P. (2011). A classification of ductal plate malformations based on distinct pathogenic mechanisms of biliary dysmorphogenesis. Hepatology.

[80] Tanimizu N., Miyajima A., Mostov K.E. (2009). Liver progenitor cells fold up a cell monolayer into a double-layered structure during tubular morphogenesis. Mol. Biol. Cell.

[81] Tanimizu N., Miyajima A., Mostov K.E. (2007). Liver progenitor cells develop cholangiocyte-type epithelial polarity in three-dimensional culture. Mol. Biol. Cell.

[82] Raynaud P., Carpentier R., Antoniou A., Lemaigre F.P. (2011). Biliary differentiation and bile duct morphogenesis in development and disease. Int. J. Biochem. Cell Biol..

[83] Gunay-Aygun M. (2009). Liver and kidney disease in ciliopathies. Am. J. Med. Genet. C Semin. Med. Genet..

[84] Kettunen J.L., Parviainen H., Miettinen P.J., Färkkilä M., Tamminen M., Salonen P., Lantto E., Tuomi T. (2017). Biliary Anomalies in Patients With HNF1B Diabetes. J. Clin. Endocrinol. Metab..

[85] Gonc E.N., Ozturk B.B., Haldorsen I.S., Molnes J., Immervoll H., Ræder H., Molven A., Søvik O., Njølstad P.R. (2012). HNF1B mutation in a Turkish child with renal and exocrine pancreas insufficiency, diabetes and liver disease. Pediatr. Diabetes.

[86] Francis Y., Tiercelin C., Alexandre-Heyman L., Larger E., Dubois-Laforgue D. (2022). HNF1B-MODY Masquerading as Type 1 Diabetes: A Pitfall in the Etiological Diagnosis of Diabetes. J. Endocr. Soc..

[87] Ayoub M.D., Kamath B.M. (2020). Alagille Syndrome: Diagnostic Challenges and Advances in Management. Diagnostics.

[88] Ibrahim S.H., Kamath B.M., Loomes K.M., Karpen S.J. (2022). Cholestatic liver diseases of genetic etiology: Advances and controversies. Hepatology.

[89] Li J.Q., Lu Y., Qiu Y.L., Wang J.S. (2018). Neonatal sclerosing cholangitis caused by DCDC2 variations in two siblings and literature review. Zhonghua Er Ke Za Zhi.

[90] Grammatikopoulos T., Sambrotta M., Strautnieks S., Foskett P., Knisely A.S., Wagner B., Deheragoda M., Starling C., Mieli-Vergani G., Smith J. (2016). Mutations in DCDC2 (doublecortin domain containing protein 2) in neonatal sclerosing cholangitis. J. Hepatol..

[91] Girard M., Bizet A.A., Lachaux A., Gonzales E., Filhol E., Collardeau-Frachon S., Jeanpierre C., Henry C., Fabre M., Viremouneix L. (2016). DCDC2 Mutations Cause Neonatal Sclerosing Cholangitis. Hum. Mutat..

[92] Khanna R., Verma S.K. (2018). Pediatric hepatocellular carcinoma. World J. Gastroenterol..

[93] Atra A., Al-Asiri R., Wali S., Al-Husseini H., Al-Bassas A., Zimmermann A. (2007). Hepatocellular carcinoma, syncytial giant cell: A novel variant in children: A case report. Ann. Diagn. Pathol..

[94] Pinon M., Gambella A., Giugliano L., Chiadò C., Kalantari S., Bracciamà V., Deaglio S., Tinti D., Peruzzi L., Cotti R. (2022). New case of syncytial giant-cell variant of hepatocellular carcinoma in a pediatric patient with HNF1B deficiency: Does it fit with the syndrome?. BMJ Open Gastroenterol..

[95] Gambella A., Mastracci L., Caporalini C., Francalanci P., Mescoli C., Ferro J., Alaggio R., Grillo F. (2022). Not only a small liver—The pathologist’s perspective in the pediatric liver transplant setting. Pathologica.

[96] Kakos C.D., Ziogas I.A., Demiri C.D., Esagian S.M., Economopoulos K.P., Moris D., Tsoulfas G., Alexopoulos S.P. (2022). Liver Transplantation for Pediatric Hepatocellular Carcinoma: A Systematic Review. Cancers.

[97] Varol F.I. (2020). Pediatric Hepatocellular Carcinoma. J. Gastrointest. Cancer.

[98] Torbenson M., Zen Y., Yeh M.M. (2018). Tumors of the Liver: American Registry of Pathology.

[99] Colclough K., Ellard S., Hattersley A., Patel K. (2022). Syndromic Monogenic Diabetes Genes Should Be Tested in Patients with a Clinical Suspicion of Maturity-Onset Diabetes of the Young. Diabetes.

[100] Saint-Martin C., Bouvet D., Bastide M., Bellanne-Chantelot C. (2022). Gene Panel Sequencing of Patients with Monogenic Diabetes Brings to Light Genes Typically Associated with Syndromic Presentations. Diabetes.

[101] Bingham C., Hattersley A.T. (2004). Renal cysts and diabetes syndrome resulting from mutations in hepatocyte nuclear factor-1beta. Nephrol. Dial. Transplant..

[102] Adalat S., Bockenhauer D., Ledermann S.E., Hennekam R.C., Woolf A.S. (2010). Renal malformations associated with mutations of developmental genes: Messages from the clinic. Pediatr. Nephrol..

[103] Tudorache E., Sellier-Leclerc A.L., Lenoir M., Toubiana N., Bensman A., Bellanne-Chantelot C., Ulinski T. (2012). Childhood onset diabetes posttransplant in a girl with TCF2 mutation. Pediatr. Diabetes.

[104] Zuber J., Bellanné-Chantelot C., Carette C., Canaud G., Gobrecht S., Gaha K., Mallet V., Martinez F., Thervet E., Timsit J. (2009). HNF1B-related diabetes triggered by renal transplantation. Nat Rev Nephrol..

[105] Lopes A.M., Teixeira S. (2021). New-onset diabetes after kidney transplantation revealing HNF1B-associated disease. Endocrinol. Diabetes Metab. Case Rep..

[106] Faguer S., Esposito L., Casemayou A., Pirson Y., Decramer S., Cartery C., Hazzan M., Garrigue V., Roussey G., Cointault O. (2016). Calcineurin Inhibitors Downregulate HNF-1beta and May Affect the Outcome of HNF1B Patients After Renal Transplantation. Transplantation.

[107] Pearson E.R., Badman M.K., Lockwood C.R., Clark P.M., Ellard S., Bingham C., Hattersley A.T. (2004). Contrasting diabetes phenotypes associated with hepatocyte nuclear factor-1alpha and -1beta mutations. Diabetes Care.

[108] Hattersley A.T., Greeley S.A., Polak M., Rubio-Cabezas O., Njølstad P.R., Mlynarski W., Castano L., Carlsson A., Raile K., Chi D.V. (2018). ISPAD Clinical Practice Consensus Guidelines 2018: The diagnosis and management of monogenic diabetes in children and adolescents. Pediatr. Diabetes.

[109] Carrillo E., Lomas A., Pines P.J., Lamas C. (2017). Long-lasting response to oral therapy in a young male with monogenic diabetes as part of *HNF1B*-related disease. Endocrinol. Diabetes Metab. Case Rep..

[110] Haumaitre C., Barbacci E., Jenny M., Ott M.O., Gradwohl G., Cereghini S. (2005). Lack of TCF2/vHNF1 in mice leads to pancreas agenesis. Proc. Natl. Acad. Sci. USA.

[111] Maestro M.A., Boj S.F., Luco R.F., Pierreux C.E., Cabedo J., Servitja J.M., German M.S., Rousseau G.G., Lemaigre F.P., Ferrer J. (2003). Hnf6 and Tcf2 (MODY5) are linked in a gene network operating in a precursor cell domain of the embryonic pancreas. Hum. Mol. Genet..

[112] Haumaitre C., Fabre M., Cormier S., Baumann C., Delezoide A.L., Cereghini S. (2006). Severe pancreas hypoplasia and multicystic renal dysplasia in two human fetuses carrying novel HNF1beta/MODY5 mutations. Hum. Mol. Genet..

[113] Haldorsen I.S., Vesterhus M., Raeder H., Jensen D.K., Søvik O., Molven A., Njølstad P.R. (2008). Lack of pancreatic body and tail in HNF1B mutation carriers. Diabet. Med..

[114] Vesterhus M., Raeder H., Johansson S., Molven A., Njolstad P.R. (2008). Pancreatic exocrine dysfunction in maturity-onset diabetes of the young type 3. Diabetes Care.

[115] Edghill E.L., Oram R.A., Owens M., Stals K.L., Harries L.W., Hattersley A.T., Ellard S., Bingham C. (2008). Hepatocyte nuclear factor-1beta gene deletions--a common cause of renal disease. Nephrol. Dial. Transplant..

[116] Edghill E.L., Bingham C., Slingerland A.S., Minton J.A.L., Noordam C., Ellard S., Hattersley A.T. (2006). Hepatocyte nuclear factor-1 beta mutations cause neonatal diabetes and intrauterine growth retardation: Support for a critical role of HNF-1beta in human pancreatic development. Diabet. Med..

[117] Janky R.S., Binda M.M., Allemeersch J., Govaere O., Swinnen J.V., Roskams T., Aerts S., Topal B. (2016). Prognostic relevance of molecular subtypes and master regulators in pancreatic ductal adenocarcinoma. BMC Cancer.

[118] Subramanian A., Tamayo P., Mootha V.K., Mukherjee S., Ebert B.L., Gillette M.A., Paulovich A., Pomeroy S.L., Golub T.R., Lander E.S. (2005). Gene set enrichment analysis: A knowledge-based approach for interpreting genome-wide expression profiles. Proc. Natl. Acad. Sci. USA.

[119] Kondratyeva L.G., Chernov I.P., Zinovyeva M.V., Kopantzev E.P., Sverdlov E.D. (2017). Expression of master regulatory genes of embryonic development in pancreatic tumors. Dokl. Biochem. Biophys..

[120] Kato H., Tateishi K., Fujiwara H., Nakatsuka T., Yamamoto K., Kudo Y., Hayakawa Y., Nakagawa H., Tanaka Y., Ijichi H. (2022). MNX1-HNF1B Axis Is Indispensable for Intraductal Papillary Mucinous Neoplasm Lineages. Gastroenterology.

[121] Roy N., Malik S., Villanueva K.E., Urano A., Lu X., Von Figura G., Seeley E.S., Dawson D.W., Collisson E.A., Hebrok M. (2015). Brg1 promotes both tumor-suppressive and oncogenic activities at distinct stages of pancreatic cancer formation. Genes Dev..

[122] Elliott K.S., Zeggini E., McCarthy M.I., Gudmundsson J., Sulem P., Stacey S.N., Thorlacius S., Amundadottir L., Grönberg H., Xu J. (2010). Evaluation of association of HNF1B variants with diverse cancers: Collaborative analysis of data from 19 genome-wide association studies. PLoS ONE.

[123] Thomson E., Tran M., Robevska G., Ayers K., van der Bergen J., Gopalakrishnan Bhaskaran P., Haan E., Cereghini S., Vash-Margita A., Margetts M. (2022). Functional genomics analysis identifies loss of HNF1B function as a cause of Mayer-Rokitansky-Kuster-Hauser syndrome. Hum. Mol. Genet..

[124] Lindner T.H., Njolstad P.R., Horikawa Y., Bostad L., Bell G.I., Sovik O. (1999). A novel syndrome of diabetes mellitus, renal dysfunction and genital malformation associated with a partial deletion of the pseudo-POU domain of hepatocyte nuclear factor-1beta. Hum. Mol. Genet..

[125] Oram R.A., Edghill E.L., Blackman J., Taylor M.J., Kay T., Flanagan S.E., Ismail-Pratt I., Creighton S.M., Ellard S., Hattersley A.T. (2010). Mutations in the hepatocyte nuclear factor-1beta (HNF1B) gene are common with combined uterine and renal malformations but are not found with isolated uterine malformations. Am. J. Obstet. Gynecol..

[126] Haeri S., Devers P.L., Kaiser-Rogers K.A., Moylan V.J., Torchia B.S., Horton A.L., Wolfe H.M., Aylsworth A.S. (2010). Deletion of hepatocyte nuclear factor-1-beta in an infant with prune belly syndrome. Am. J. Perinatol..

[127] Moreno-De-Luca D., Mulle J.G., Kaminsky E.B., Sanders S.J., Myers S.M., Adam M.P., Pakula A.T., Eisenhauer N.J., Uhas K., Weik L. (2010). Deletion 17q12 is a recurrent copy number variant that confers high risk of autism and schizophrenia. Am. J. Hum. Genet..

[128] Loirat C., Bellanne-Chantelot C., Husson I., Deschenes G., Guigonis V., Chabane N. (2010). Autism in three patients with cystic or hyperechogenic kidneys and chromosome 17q12 deletion. Nephrol. Dial. Transplant..

[129] Nagamani S.C.S., Erez A., Shen J., Li C., Roeder E., Cox S., Karaviti L., Pearson M., Kang S.H.L., Sahoo T. (2010). Clinical spectrum associated with recurrent genomic rearrangements in chromosome 17q12. Eur. J. Hum. Genet..

[130] Mefford H.C., Clauin S., Sharp A.J., Moller R.S., Ullmann R., Kapur R., Pinkel D., Cooper G.M., Ventura M., Ropers H.H. (2007). Recurrent reciprocal genomic rearrangements of 17q12 are associated with renal disease, diabetes, and epilepsy. Am. J. Hum. Genet..

[131] Quintero-Rivera F., Woo J.S., Bomberg E.M., Wallace W.D., Peredo J., Dipple K.M. (2014). Duodenal atresia in 17q12 microdeletion including HNF1B: A new associated malformation in this syndrome. Am. J. Med. Genet. A.

[132] Murray P.J., Thomas K., Mulgrew C.J., Ellard S., Edghill E.L., Bingham C. (2008). Whole gene deletion of the hepatocyte nuclear factor-1beta gene in a patient with the prune-belly syndrome. Nephrol. Dial. Transplant..

[133] Hendrix N.W., Clemens M., Canavan T.P., Surti U., Rajkovic A. (2012). Prenatally diagnosed 17q12 microdeletion syndrome with a novel association with congenital diaphragmatic hernia. Fetal Diagn. Ther..

[134] Hinkes B., Hilgers K.F., Bolz H.J., Goppelt-Struebe M., Amann K., Nagl S., Bergmann C., Rascher W., Eckardt K.U., Jacobi J. (2012). A complex microdeletion 17q12 phenotype in a patient with recurrent de novo membranous nephropathy. BMC Nephrol..

[135] Faguer S., Chassaing N., Bandin F., Prouheze C., Garnier A., Casemayou A., Huart A., Schanstra J.P., Calvas P., Decramer S. (2014). The HNF1B score is a simple tool to select patients for HNF1B gene analysis. Kidney Int..

